# An assessment of heavy metals in green sea turtle (*Chelonia mydas*) hatchlings from Saudi Arabia’s largest rookery, Ras Baridi

**DOI:** 10.7717/peerj.13928

**Published:** 2022-08-23

**Authors:** Lyndsey K. Tanabe, Kirsty Scott, Vijayalaxmi Dasari, Michael L. Berumen

**Affiliations:** Red Sea Research Center, Division of Biological and Environmental Science and Engineering, King Abdullah University of Science and Technology, Thuwal, Saudi Arabia

**Keywords:** Sea turtle, Heavy metal, Pollution, Contamination, Red Sea, Saudi Arabia, *Chelonia mydas*, Cement, Conservation

## Abstract

**Background:**

Anthropogenic sources can lead to the accumulation of heavy metals in marine organisms through ingestion, absorption, or inhalation. For sea turtle embryos, heavy metals can be absorbed into the egg from the incubation environment or be maternally transferred to the offspring causing neurological, reproductive, and developmental problems. Here, we report heavy metal concentrations in green turtle hatchlings from the largest rookery on the Red Sea, Ras Baridi.

**Methods:**

Deceased hatchlings were collected from two beaches near a cement factory at Ras Baridi, from which heavy metal concentrations (chromium (Cr), manganese (Mn), iron (Fe), cobalt (Co), nickel (Ni), copper (Cu), zinc (Zn), arsenic (As), selenium (Se), cadmium (Cd), and lead (Pb)) were measured from the liver, muscle, and residual yolk of the hatchlings.

**Results:**

Although based on a small sample of hatchlings, the data presented here provides the first measurements of heavy metals from sea turtles in the Red Sea and highlights the link between human activity and its impact on the ecology of sea turtles. In general, the heavy metal concentrations of heavy metals were not significantly different between the beach next to the cement factory and the beach downwind from the factory. However, the concentrations of heavy metals were significantly different between sampled tissues (liver, muscle, and residual yolk).

**Discussion:**

This study provides insight into current heavy metal levels in green turtle hatchlings, which can be used as bio-indicators for environmental contaminants as coastal development increases in the Red Sea. Moreover, we found a lack of standardized methodology to evaluate heavy metals in hatchling sea turtles. Future efforts should work toward creating comparable techniques for long-term heavy metal monitoring, as this is a useful determinant of anthropogenic pollution.

## Introduction

The green sea turtle, *Chelonia mydas*, is classified as Endangered by the International Union on Conservation of Nature (IUCN, 2022). This conservation status is largely caused by incidental bycatch, direct poaching, entanglement, and boat strikes (Stanford et al., 2020). Additionally, anthropogenic activities generate environmental pollutants of concern due to their potential toxicity to marine fauna. For example, green sea turtles can be infected with a disease known as fibropapillomatosis (FP) which causes benign tumors that can grow large enough to inhibit sight, mobility, and foraging (Herbst, 1994). It is hypothesized that environmental contamination, including those from heavy metals, may be a cofactor (da Silva et al., 2014). In addition, sea turtles exposed to high concentrations of environmental pollutants, particularly heavy metals, have been shown to develop neurological and developmental disorders, cancer, and in more severe cases, death (Marsili & Fossi, 2003; Nordberg et al., 2007; Grillitsch & Schiesari, 2010). Since sea turtles are long-lived vertebrates with large home ranges, they are susceptible to the accumulation of heavy metals in their tissues (Sakai et al., 1995); because of this, they are often used as bio-indicators for environmental contaminants (Bruno et al., 2021). Sea turtle eggs, embryos, and hatchlings are vulnerable to heavy metal contamination as contaminants bioaccumulated in the female can be maternally transferred to the embryo and absorbed from the nest environment during incubation (Al-Rawahy et al., 2007; Hopkins, Willson & Hopkins, 2013). Sea turtle eggs incubate for 2 months, allowing sufficient time for contaminants to be absorbed into the developing embryo (Bustard & Greenham, 1968; Ackerman & Prange, 1972). Environmental contaminants can have significant impacts on embryotic development and can potentially contribute to embryo mortality (Hamlin & Guillette, 2010). Defects from these pollutants (*i.e*., thyroid dysfunction, reduced hatching success, eggshell thinning, and other abnormalities) are a potential source of mortality for turtle embryos, preventing survival to their juvenile life stage (Hamlin & Guillette, 2010).

In the past three decades, industrial activity and development have increased in Saudi Arabia’s coastal areas, introducing hazardous elements into the environment (Youssef & El-Sorogy, 2016; Al-Mur, Quicksall & Al-Ansari, 2017). In addition, rapid human population growth and urbanization have further increased pollution (Ozturk & Turkan, 1993; Badr et al., 2009). This is of particular concern in the region, as the population growth rate around the Red Sea is expected to double in the next 20–30 years (United Nations, 2017) with the development of large-scale “giga-projects” under the Kingdom’s Vision 2030 (PIF, 2017). Heavy metals may be introduced into the environment by aeolian transport (Olsen, Cutshall & Larsen, 1982) or directly from industrial pollution. This leads to their integration into sediments and the water column, where they are ultimately absorbed by living organisms (Olsen, Cutshall & Larsen, 1982; Claereboudt, 2004). Among these hazardous pollutants are elements with high molecular weight, such as chromium (Cr), manganese (Mn), iron (Fe), cobalt (Co), nickel (Ni), copper (Cu), zinc (Zn), arsenic (As), selenium (Se), cadmium (Cd), and lead (Pb). In high concentrations, these elements can have detrimental impacts on marine organisms (Khansari, Ghazi-Khansari & Abdollahi, 2005). Furthermore, there are additional environmental concerns as these pollutants do not biodegrade naturally (Briffa, Sinagra & Blundell, 2020). Despite reports suggesting sea turtles are considerably affected by heavy metal pollution (Lutcavage, Plotkin & Witherington, 1997; Sakai et al., 2000; Al-Rawahy et al., 2007), the majority of the work on heavy metal pollution to date has focused on salt marshes, estuaries, and mangrove systems.

Cement production is a significant source of heavy metal pollution (Achternbosch, Bräutigam & Gleis, 2003). Heavy metals such as Cd, Cr, Cu, Pb, and Zn are used in cement production and can be toxic to humans and animals, even at low concentrations (Al-Khashman & Shawabkeh, 2006; Kabata-Pendias & Mukherjee, 2007). The concentration of the heavy metals deposited varies depending on the wind velocity and particle size through the cement dust (Central Pollution Control Board (CPBC), 2007). The main transport pathway is via wind flowing over the cement kilns and releasing heavy metals into the air or settling as dust (Central Pollution Control Board (CPBC), 2007). In Oman, high concentrations of heavy metals have been recorded within a 0.5 and 2 km radius around a cement factory, affecting surrounding biota (Semhi et al., 2010). The severity of heavy metal pollution is dictated by the accumulation pathway, pollutant type, and vulnerability of the species exposed (Adekola, Inyinbor & Abdul Raheem, 2012).

Ras Baridi is the largest rookery for green turtles in the Red Sea, with an estimated 250 females nesting annually (Pilcher & Al-Merghani, 2000; Shimada et al., 2021). Ras Baridi is ~50 km north of the city of Yanbu and is located adjacent to the Yanbu Cement Factory. It has been proposed that cement dust from the kilns is blown onto the adjacent nesting beach, which compacts the sand, preventing hatchling emergence (Pilcher, 1999). Despite the potential negative impacts of cement dust at Ras Baridi first revealed 20 years ago (Pilcher, 1999), there have been no subsequent studies on the influence of the cement dust on incubating embryos on this nesting beach. Physical and environmental parameters of this nesting beach have been studied, including the sedimentology (Al-Mansi, Nawab & Sagga, 1991; Scott et al., 2022), temperature (Tanabe et al., 2020), sand compaction (Pilcher, 1999) and heavy metal concentrations in the sand (Tanabe et al., 2022) but heavy metal accumulation in sea turtle hatchlings is yet to be examined, with most studies worldwide conducted on stranded adults of this species (Bruno et al., 2021).

The bioaccumulation of heavy metals in green turtles has been identified for a number of populations (Godley, Thompsonà & Furnessà, 1999; Sakai et al., 2000; Lam et al., 2006), but to date, there have not been any studies conducted on Saudi Arabian turtle populations. As a result, information on the heavy metal biorisk in the Red Sea is limited. The present study was conducted at Ras Baridi to assess the heavy metal concentrations in the liver, muscle, and residual yolk in green turtle hatchlings to provide a baseline and the first report of these measurements in the Red Sea region. This study is a first step toward determining the impact of heavy metal contamination on endangered sea turtle populations in the Red Sea.

## Methods

**Figure 1:**
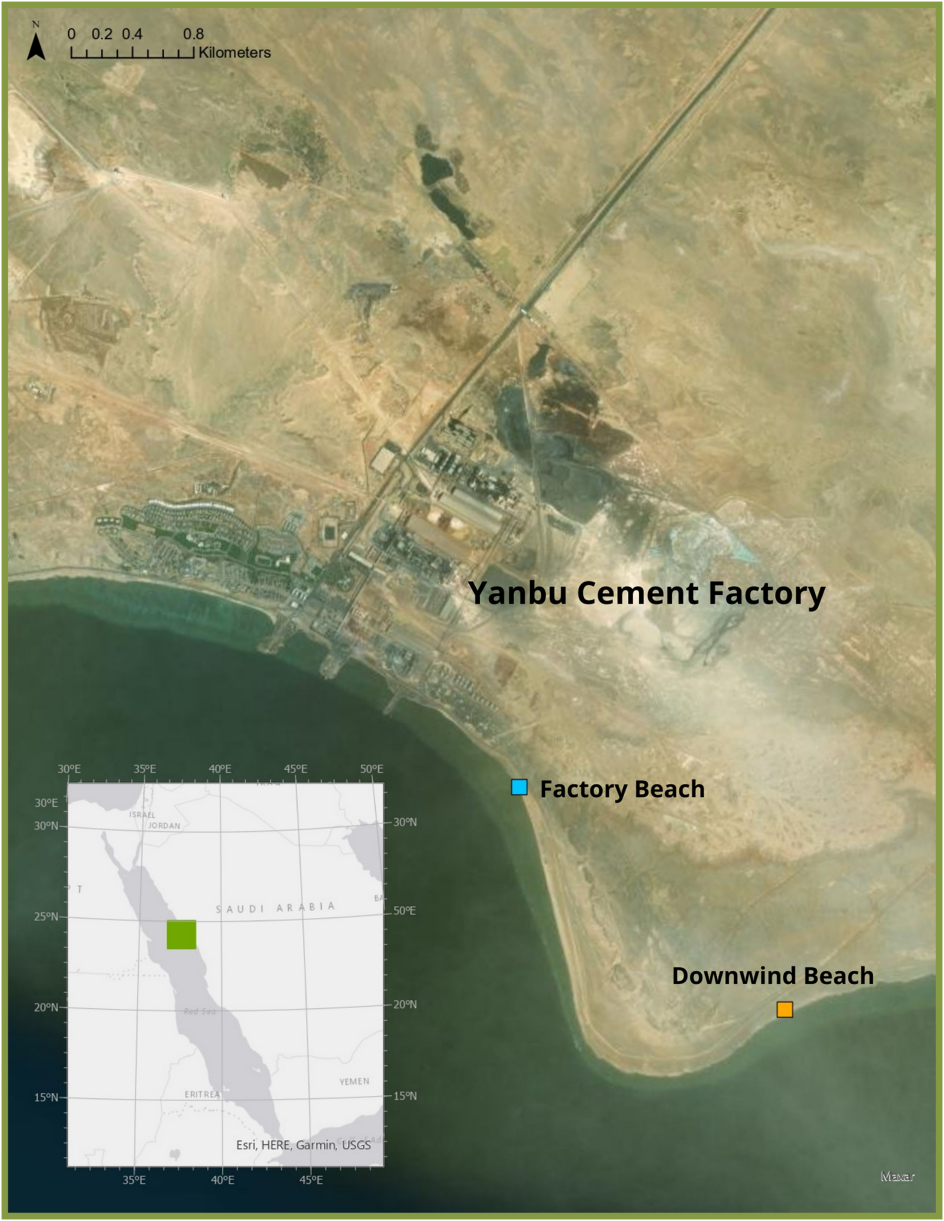
Study Site where deceased hatchlings were collected at Ras Baridi. Ras Baridi, a 6 km long stretch of coastline composed of five green turtle (*Chelonia mydas*) nesting beaches located on the Saudi Arabian Red Sea. Ras Baridi is situated adjacent to the Yanbu Cement Factory, which potentially emits dust that accumulates on the beaches downwind. Deceased hatchlings were collected from a beach next to the factory (blue), and the beach 1.5 km south (downwind) from the factory (orange). Source: ESRI, HERE, Garmin, USGS, Maxar.

Freshly deceased green sea turtle hatchlings were collected in September 2021 from two beaches in Ras Baridi (24.258°, 37.571°). These turtles were presumed dead from the night prior to collection as they were still soft and had not been consumed by predators. The cause of death of these hatchlings is not known, but it is likely they emerged from their nest and were disoriented from the lights of the cement factory and were unable to make it to the sea by sunrise, eventually dying from heat exposure. Six hatchlings were collected from the beach adjacent to the cement factory (Fig. 1, blue), and 16 were collected from the beach ~1.5 km south (downwind) of the factory (Fig. 1, orange). In the summer in Ras Baridi, the prevailing wind direction is from North to South (Langodan et al., 2017). The GPS coordinates were recorded at the collection locations, and the hatchlings were immediately frozen for transport and stored at −20 °C until analysis. The hatchlings were weighed using a Mettler Toledo XS205 Dual Range Balance (accuracy ± 0.71 mg), and their straight carapace length and straight carapace width were measured with a Dual Reading Dial Caliper (Anytime Tools, accuracy ± 0.02 mm). This fieldwork was approved by King Abdullah University of Science and Technology who obtained coast guard permits for the site. Although no work with live animals was conducted in this study, the research team has approval from KAUST’s IACUC to work with turtles under protocol 19IACUC07.

Hatchlings were dissected, and a minimum of 0.1 g of liver, muscle, and residual yolk tissue were obtained from each individual. All tissue samples were weighed for wet mass and subsequently dried in a Binder incubator at 60 °C for 3 days. The samples were then re-weighed to measure the dry mass before homogenizing using a porcelain mortar and pestle. Each ground sample was digested with a mixture of 1.5 ml of 69% nitric acid (HNO_3_) and 0.4 ml of 30% hydrogen peroxide (H_2_O_2_) using an Ultrawave digestive system (Table S1, also see Supplementary Methods for standard preparations). A minimum dry weight of 50 mg was attained by combining samples from the same location to ensure sufficient material was available for analyses (Table S2). The samples were left to cool before being diluted with Milli-Q water, and elemental analysis was quantified using an Agilent 8800 Inductively Coupled Plasma Mass Spectrometer (ICP-MS). The accuracy of the method was tested by analyzing a Certified Testing Material (ERM-CE278K) from European Reference Materials (Table S3). Quality controls were applied during the metal analysis including calibration blanks, matrix reaction blanks, continuous calibration verification, second vendor verification, duplicate samples, spiked blanks, and spiked samples once every 20 samples (Table S4). The concentration of Cr, Mn, Fe, Co, Ni, Cu, Zn, As, Se, Cd, and Pb was measured for each sample. These elements were chosen to mirror hatchling studies from other parts of the world (Stoneburner, Nicora & Blood, 1980; Sakai et al., 1995; Kaska & Furness, 2001).

All statistical analyses were conducted on R Studio Version 2021.09.0 (R Core Team, 2021), and statistical significance was assumed at *p* < 0.05. A Levene’s test and a Shapiro-Wilk were used to confirm equal variance and normality. Because some of the data did not meet the statistical prerequisites to conduct parametric tests (*i.e*., our data showed non-normal distribution), we conducted non-parametric tests. A Mann-Whitney U test was used to compare heavy metal concentrations between the sampled beaches anda Kruskal-Wallis test was used to compare heavy metal concentrations by tissue (liver, muscle, and yolk). Finally, a Spearman rank correlation coefficient was used to assess the relationship between hatchling size and mass with heavy metal concentrations. To contextualize the data, the average concentration of each heavy metal in each tissue (liver, muscle, and yolk) were descriptively compared to the average concentration of As, Cd, Cr, Cu, Fe, Ni, Pb, and Se (Tanabe et al., 2022) measured in the sand at the two sampled beaches. These sand samples were collected on May, 2021 (4 months prior to when the hatchlings were sampled).

## Results

The Mann-Whitney U test revealed that As was the only metal to demonstrate a significant difference between beaches. The concentration of As in hatchlings’ tissues measured lower on the beach adjacent to the cement factory compared to the beach downwind from the factory (W = 472, *p* < 0.01) (Table S5). Because all other metals showed no significant differences between the two sites, the concentrations from both beaches were combined and the average heavy metal concentrations (dry weight, mg/kg) in the liver, muscle, and residual yolk were reported (Table 1).

**Table 1:** Average heavy metal concentrations from liver, muscle, and yolk of deceased hatchlings.

	*n*	Cr	Mn	Fe	Co	Ni	Cu	Zn	As	Se	Cd	Pb
Liver	19	10.29 ± 8.35	3.11 ± 0.82	423.58 ± 111.89	0.11 ± 0.08	5.60 ± 5.05	10.41 ± 9.77	36.57 ± 6.51	0.55 ± 0.75	2.53 ± 0.72	0.01 ± 0.02	0.09 ± 0.11
Muscle	10	20.18 ± 15.97	3.28 ± 1.03	280.47 ± 86.82	0.21 ± 0.16	11.41 ± 9.00	4.78 ± 2.23	95.85 ± 22.86	0.90 ± 1.05	0.17 ± 0.31	0.01 ± 0.02	0.24 ± 0.23
Residual yolk	22	2.66 ± 1.65	0.65 ± 0.25	43.72 ± 27.31	0.05 ± 0.03	1.61 ± 1.04	1.62 ± 0.54	56.12 ± 46.33	0.48 ± 0.63	1.63 ± 0.56	0.11 ± 0.24	0.05 ± 0.03

**Note:**

Average heavy metal concentrations (dry weight, mg/kg) ± standard deviation measured from internal tissues (liver, muscle, and residual yolk) of green turtles (*Chelonia mydas*) hatchlings from Ras Baridi, Saudi Arabia. The sample size included in analyses is found in column “*n*”. Heavy metals measured include chromium (Cr), manganese (Mn), iron (Fe), cobalt (Co), nickel (Ni), copper (Cu), zinc (Zn), arsenic (As), selenium (Se), cadmium (Cd), and lead (Pb).

Iron from the liver of hatchlings presented a maximum measurement of 630.8 mg/kg and an overall mean of 423.58 ± 111.89 mg/kg (Table 1). The concentration of Fe in the muscle was also high, averaging 280.47 ± 86.82 mg/kg. The mean concentration of Fe in the yolk was much lower at 43.72 ± 27.31 mg/kg. In the yolk, Zn exhibited the highest levels of all elements, with an average concentration of 56.12 ± 46.33 mg/kg (Table 1). Cd had the lowest average concentrations compared to the other elements, with the smallest concentration measured in the liver (0.01 ± 0.02 mg/kg) (Table 1).

**Figure 2:**
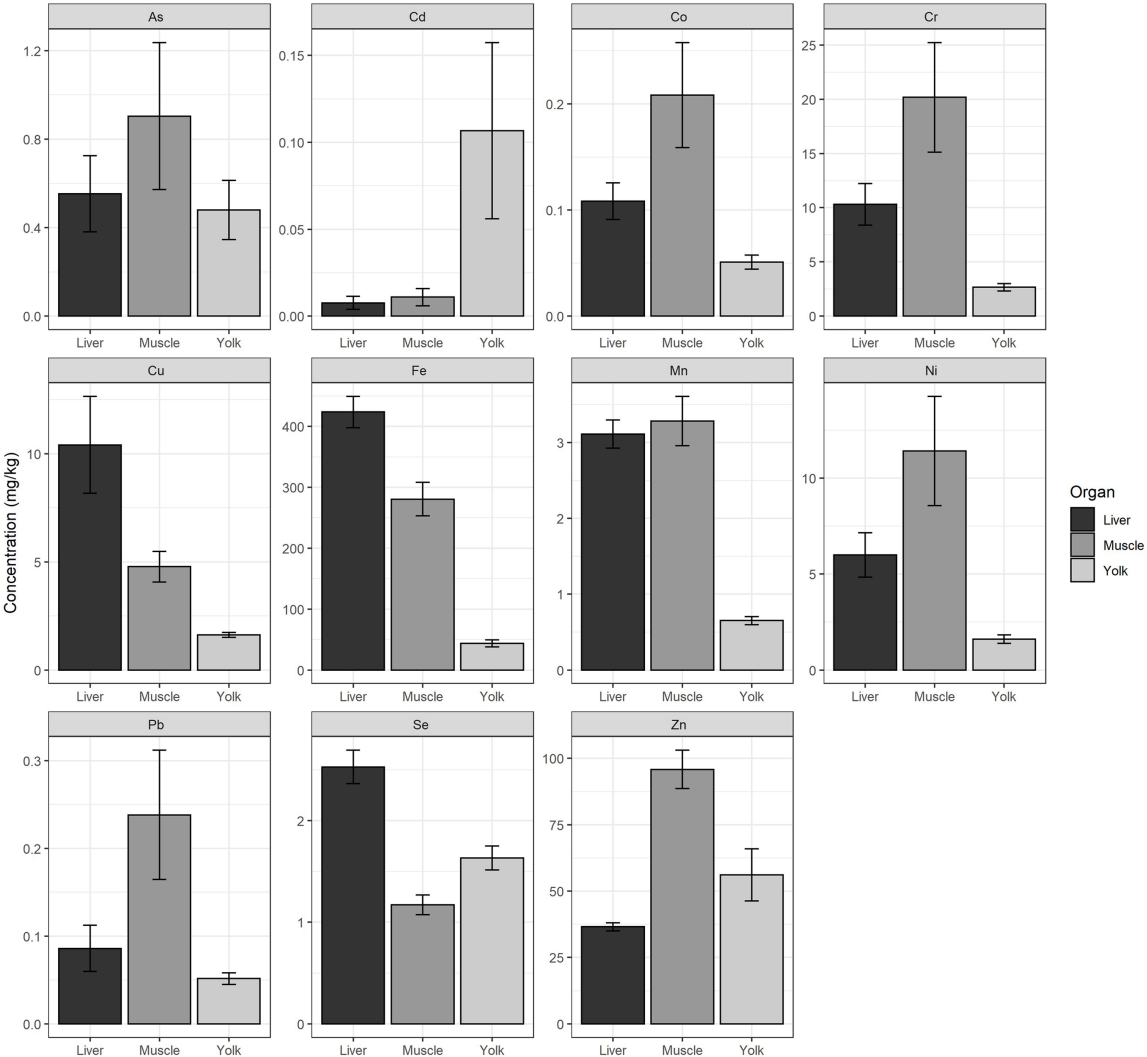
Average heavy metal concentrations from liver, muscle, and yolk of hatchlings. Average heavy metal concentrations (dry weight, mg/kg) ± standard error (se) of arsenic (As), cadmium (Cd), cobalt (Co), chromium (Cr), copper (Cu), iron (Fe), manganese (Mn), nickel (Ni), lead (Pb), selenium (Se), and zinc (Zn) from the liver, muscle, and yolk of 22 green turtle (*Chelonia mydas*) hatchlings collected at Ras Baridi. Concentrations are shown on the y-axis (note varying scale for each element).

We found significant differences in the concentrations of each heavy metal (Cr, Mn, Fe, Co, Ni, Cu, Zn, As, Se, Cd, and Pb) measured between hatchlings’ tissue (yolk, liver, and muscle) (*p* < 0.05, Table S6). We found that the yolk had the highest concentration of Cd. The liver had the highest concentrations of Cu, Fe, and Se, and the muscle had the highest concentrations of As, Co, Cr, Mn, Ni, Pb, and Zn (Fig. 2).

Lastly, we found no significant correlation between overall heavy metal concentration and hatchling mass (*r*(241) = 0.0914, *p* = 0.1563) or carapace length (*r*(241) = 0.0649, *p* = 0.3144). Though, when correlation was tested for each element separately, the only significant result found was Cd, which showed a negative correlation for both mass (*r*(43) = −0.6, *p* = 0.004) and carapace length (*r*(43) = −0.45, *p* = 0.036).

**Figure 3:**
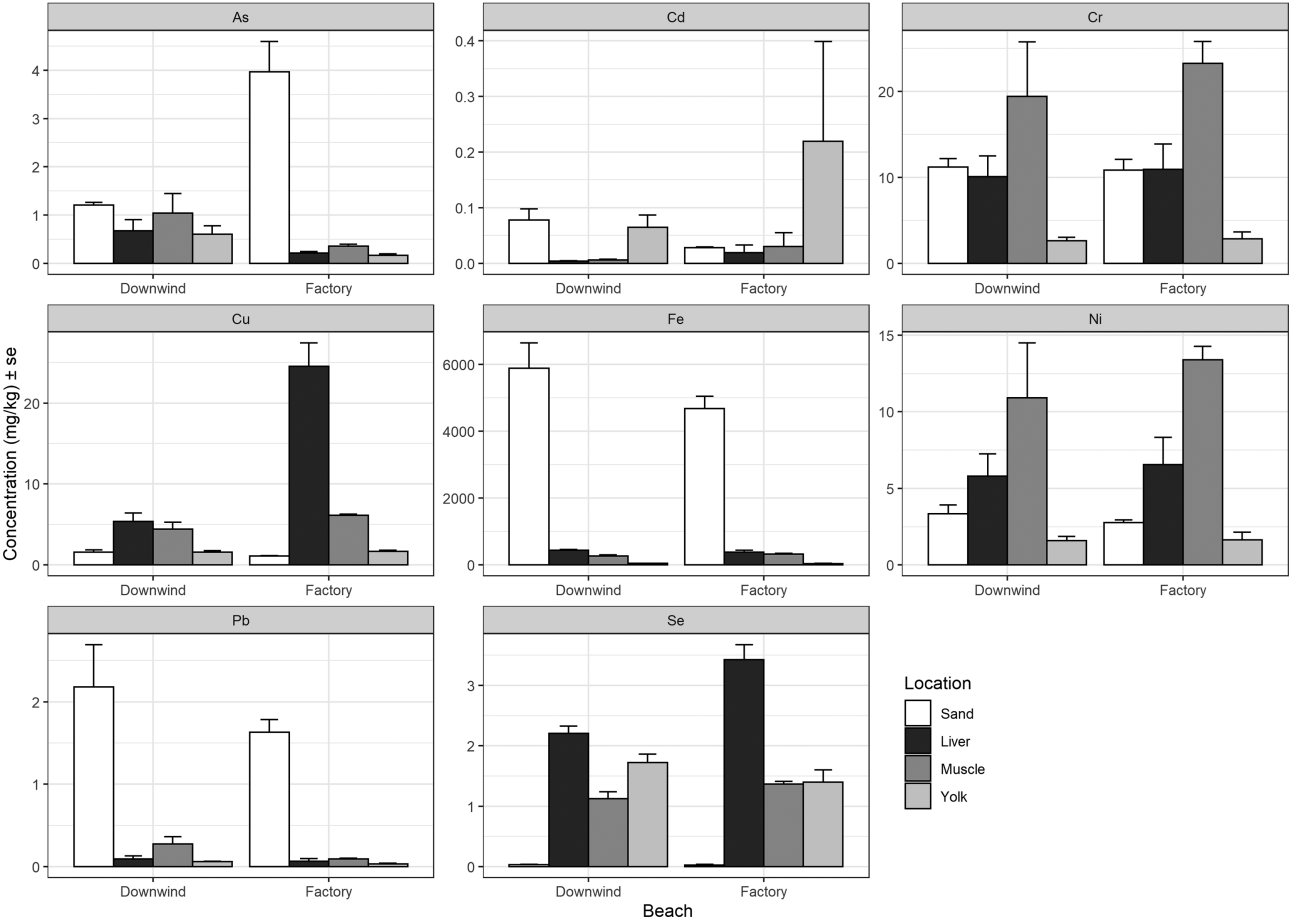
Heavy metal comparison between sand at nesting beach and hatchlings’ tissues. Average heavy metal concentrations (dry weight, mg/kg) + standard error (se) of arsenic (As), cadmium (Cd), chromium (Cr), copper (Cu), iron (Fe), nickel (Ni), lead (Pb), and selenium (Se) from the sand at the nesting beaches (Tanabe et al., 2022) compared to the liver, muscle, and residual yolk of the green turtle (*Chelonia mydas)* hatchlings from the beach next to the Ras Baridi cement factory and the beach 1.5 km downwind from the factory. Concentrations are shown on the y-axis (note varying scale for each element).

There was no consistent relationship found between heavy metal concentrations measured in the nesting environment to the concentrations measured in each tissue (liver, muscle, and residual yolk) (Fig. 3). Arsenic demonstrated higher concentrations in the sand at the site adjacent to the factory compared to the beach downwind, but this pattern was reversed in the tissues of the hatchlings (Fig. 3). Cadmium showed a similar result, but the concentration at the site downwind was higher than at the beach next to the factory, whereas the pattern was reversed in the tissues of the hatchlings (Fig. 3). Chromium and Ni had similar results to each other, which generally had lower measured sand concentrations than measured in the liver and muscle, but higher than in yolk (Fig. 3). Copper and Se concentrations in hatchlings’ tissues do not seem to have a relationship with the nest environment, as the mean concentration in the sand was lower than the mean concentration measured in the yolk, muscle, and liver (Fig. 3). Finally, Fe and Pb were much higher in the sand at these beaches compared to in the hatchlings’ tissues.

## Discussion

Our findings provide the first measurements of heavy metals from green turtles in the Red Sea. We found no significant differences in heavy metal concentrations in hatchlings collected on the nesting beach adjacent to the cement factory compared to the beach 1.5 km downwind, with the exception of As. Furthermore, we found significant differences in heavy metal concentrations among hatchlings’ tissues (liver, muscle, and residual yolk). Seven of the heavy metals were measured highest in the muscle, three were highest in the liver, and only Cd was highest in the yolk. Also, we did not find many significant correlations between the heavy metal concentrations with the hatchlings’ mass or carapace length for most metals. There were no clear trends between the concentration of heavy metals measured in the hatchlings’ tissue and the sand at the beaches where they were found, but Fe and Pb potentially demonstrated a relationship between the sand and hatchlings’ tissue concentrations. Although our sample size is relatively small, these data provide an important initial assessment against which future measurements can be compared.

Arsenic was found to have significantly lower concentrations measured in the hatchlings’ tissues from the beach next to the factory compared to the beach 1.5 km downwind. When these concentrations were compared to the mean As concentration in the sand at these two beaches, there was no relationship, and the concentration in the sand was much higher at the factory compared to the site downwind (Tanabe et al., 2022). A study on As uptake by reptilian flexible-shelled eggs from contaminated nest substrates found that As concentration was significantly higher in eggshells compared to the embryos, suggesting that the eggshell may inhibit the passage of As into the eggs (Marco, López-Vicente & Pérez-Mellado, 2004).

### Inter-tissue variability of heavy metals in green turtle hatchlings

All heavy metals investigated were present in all tissues sampled in green turtle hatchlings, but the pattern of concentration among the liver, muscle, and residual yolk varied by metal. Heavy metal contamination could be due to both maternal and nesting beach substrate transfer (Sakai et al., 1995; Grillitsch & Schiesari, 2010). Cement factories are known to produce Zn, Cd, and Pb (Chen et al., 2010; Lu et al., 2016). Mean concentration of Zn measured from livers of Ras Baridi green turtle hatchlings (25.57 ± 3.10 mg/kg) was slightly higher than the mean concentration of livers of loggerhead hatchlings from Turkey (23.84 ± 3.10 mg/kg) (Kaska & Furness, 2001), while the average Zn concentration in the yolk of hatchlings was similar (Ras Baridi green hatchlings: 56.12 ± 46.33 mg/kg, Turkey loggerhead hatchlings: 57.21 ± 2.23 mg/kg) (Kaska & Furness, 2001). Studies suggest that Zn is an element that can be maternally transferred in turtles (Sakai et al., 1995; Ehsanpour et al., 2014) and has been shown to inhibit neural transmission when present in nanoparticle form (Kumar et al., 2020). Furthermore, sea turtles have been shown to accumulate Zn 10-fold compared to other marine megafauna due to the pigment in their fat (Sakai et al., 2000), meaning Zn toxicity may pose an increased threat to sea turtles. Alternatively, Cd and Pb were found in lower concentrations in both the liver and yolk of Ras Baridi green turtle hatchlings than loggerhead hatchlings from Turkey (Kaska & Furness, 2001). Both Cd and Pb are transferred from the nesting environment of turtles (Al-Rawahy et al., 2007) and maternally transferred (Ehsanpour et al., 2014; Sinaei & Bolouki, 2017). The Pb concentration in the sand measured at the nesting beaches (Tanabe et al., 2022) was much higher compared to the levels measured in the hatchlings’ tissue, which was not a pattern as obvious for Cd, which had concentration levels in the hatchlings’ yolk exceeding the concentrations measured in the sand (Fig. 3).

The elements Cr, Se, As, Mn, Co, and Ni were also detected at different levels depending on the tissue sampled, but unfortunately, there was little published data on these metals in sea turtle hatchlings that used comparable methods (*i.e*., using dry weight). Similar patterns of heavy metal concentrations were found for Cr, As, Mn, Co, and Ni, with average heavy metal concentrations lowest in the yolk, followed by the liver and the highest concentrations in the muscle. Of these heavy metals, Cr had the highest concentrations. Cr has been found to have various adverse health effects in reptiles, including cancer, reproductive and developmental disorders, immune function disorders, and renal and hepatic dysfunction (Grillitsch & Schiesari, 2010). Chromium concentrations in the sand at the nesting beach is lower or similar to concentrations measured in the hatchlings’ liver and muscle, but higher than the concentrations measured in the hatchlings’ residual yolk. Additionally, As has a large range of detrimental effects, including cancer, developmental disorders, and organ dysfunction (Grillitsch & Schiesari, 2010), while excess Mn has been shown to cause endocrine disruption and neurotoxic disorders (Grillitsch & Schiesari, 2010). Arsenic concentration in the hatchlings’ tissues did not seem to have any correlation with the concentration reported in the sand, and the concentration of Mn in the sand at the nesting beach has not been reported. Although most studies on Ni accumulation in oviparous animals have been conducted on birds, high levels of Ni are associated with malformation of the embryo (Lam et al., 2006). The adverse effects of Co are not well understood, but it is known to produce an inflammatory response and the formation of free radicals in sea turtles (Gaus et al., 2019). Cobalt accumulation has also been linked to a mass stranding event of sea turtles in 2012, caused by neurotoxicity. In addition, Co has a strong affinity to red blood cells, and considering the long lifespan of red blood cells in reptiles, there is a slower elimination period of Co for these species, thus putting them at greater risk (Mader & Divers, 2014). A different pattern of concentration was found for Se, which showed highest concentration in the liver, followed by yolk and muscle. This heavy metal is known to bioaccumulate in marine food webs and has been shown to cause embryonic deformities in various water birds (Ohlendorf et al., 1986; Hoffman, Ohlendorf & Aldrich, 1988; Hamilton, 2004). The Se concentration in the sand at the nesting beach was negligible compared to the concentrations measured in the hatchlings’ tissue (Fig. 3).

Iron had a maximum concentration of 630.8 mg/kg found in the liver of a hatchling. The mean concentration of Fe measured from the livers of green turtles at Ras Baridi (423.58 ± 111.89 mg/kg) was much higher than values measured in the livers of loggerhead embryos in Turkey (35.83 ± 9.98 mg/kg) (Kaska & Furness, 2001). Iron is a highly abundant metal in the earth’s crust (American Public Health Association, 1993), and it is an essential element for living organisms, vital for growth and development (Valko, Morris & Cronin, 2005). Despite Fe being an essential element, it can be considered toxic at high concentrations (Madiwale & Liebelt, 2006). Iron has been recorded in high concentrations in the sand at Ras Baridi (Fig. 3) (Tanabe et al., 2022); hence it is possible that Fe was absorbed during incubation; alternatively, it can be maternally transferred as foraging females can accumulate Fe through their diet and transfer these elements to their offspring (Sakai et al., 1995). However, adult green turtles in Mexico were found to have much lower average Fe levels detected in their liver (14.35 mg/kg, dry weight) and their muscle (20.99 mg/kg, dry weight) (Gardner et al., 2006). In humans, elevated body storage of Fe has been shown to increase the risk of several cancers (Stevens et al., 1988), but the effects of excess iron on turtles remain unclear. Furthermore, a study on heavy metals around green turtle nesting habitat in Turkey found the highest concentrations of Fe in seagrass, the primary food source for green turtles, compared to all the substances they measured, including seagrass, beach sand, sea and river water, sediment, and several species of plants (Çelik et al., 2006). Thus, future research should also include assessing the heavy metal concentration from the large seagrass meadows near Ras Baridi, an important foraging ground for sea turtles and dugongs (Preen, 1989; Pilcher & Al-Merghani, 2000).

Copper is considered an essential element, and had a maximum measured concentration of 30.52 mg/kg, which was found in the liver of a hatchling. However, the mean Cu concentration measured from hatchlings’ livers was 10.40 ± 9.77 mg/kg. This average was lower than from loggerhead embryos’ livers in Turkey which was 21.21 ± 2.62 mg/kg (Kaska & Furness, 2001). Conversely, the average concentration of Cu in the yolk was measured higher at Ras Baridi (43.72 ± 27.31 mg/kg) compared to the yolk of loggerhead hatchlings in Turkey (15.79 ± 2.62 mg/kg) (Kaska & Furness, 2001). These differences could be due to differential contamination of the sand or dietary or behavioral differences between the turtle species. Further, Cu has been reported to accumulate in marine algae due to its use in antifouling paints (Singh & Turner, 2009). Considering that the Red Sea is one of the most heavily trafficked waterways globally (Ship Traffic, 2022), the elevated Cu concentrations in our sample may stem from shipping-related pollution. Potential adverse effects of Cu in reptiles include immune function disorders, renal dysfunction, and hepatic dysfunction (Grillitsch & Schiesari, 2010), where it has been shown to accumulate from both the nest environment (Al-Rawahy et al., 2007) and from maternal transfer (Sakai et al., 1995; Ehsanpour et al., 2014; Sinaei & Bolouki, 2017). Though we found that the Cu concentration in the sand at the nesting beach was lower compared to that measured in the hatchlings’ tissues (Fig. 3).

### Bioaccumulation relative to sea turtle size

In general, we did not find many significant correlations between the heavy metal concentration and the hatchlings’ mass or carapace length. The one exception was Cd, in which we found a significant negative correlation. This finding is in agreement with previous studies (Gardner et al., 2006; da Silva et al., 2014). Both Cu and Cd have been found to present a negative correlation between the concentration and the curved carapace length in the muscle of stranded green turtles in Brazil (da Silva et al., 2014). One possible explanation for the negative correlation is the growth dilution effect, where contaminant concentration in the tissue would likely decrease in association with the growth of the turtles (McKenzie et al., 1999). Our sampled turtles were all neonate hatchlings, so the variation of heavy metal concentration was entirely derived from maternal transfer or from the nesting environment instead of dietary differences. This may also explain the lack of significant correlations between carapace length and other elements.

### Future research

Overall, there was no clear relationship between the concentration of heavy metals measured in the sand and the concentration measured in hatchlings’ tissues (Fig. 3). The Fe and Pb concentrations were much higher in the sand compared to in the hatchlings, which could potentially be transferred to the hatchlings, hence future studies should focus on these two elements, as well as Cr, Cu, Ni, and Zn, which were found in high concentrations in hatchlings’ tissues (Fig. 2). Furthermore, the lack of standardized methods for reporting heavy metals (*e.g*., dry weight *versus* wet weight) obstructs efforts to compare results in different regions of the world. The majority of publications use dry tissue weight as a measurement unit, but there are still a significant number that do not clearly state which method is used, making comparison difficult (Grillitsch & Schiesari, 2010; Jovičić, Lenhardt & Jarić, 2015). There are advantages and disadvantages to both methods, we recommend dry weight because of the associated errors with wet weight (Adrian & Stevens, 1979). There is also a lack of understanding of the threshold levels of heavy metals and their physiological effects on sea turtles. Thus, additional studies are needed to fully understand how the physiology of these endangered species are affected by anthropogenic contamination.

Since Ras Baridi is the largest turtle rookery in Saudi Arabia, it is vital to understand the possible anthropogenic impacts caused by the nearby cement factory to conserve the green turtle population. Cement production is known to emit several heavy metals, which can be deposited in nearby sediment at varying distances depending on particle size and wind velocity (Schuhmacher, Nadal & Domingo, 2009; Central Pollution Control Board (CPBC), 2007). Sea turtle populations and their threats are relatively understudied in the Red Sea region (PERSGA, 2004; Mancini, Elsadek & El-alwany, 2015; Phillott & Rees, 2018; Hamann et al., 2022). Future research should incorporate hatchlings from other rookeries as well as a nesting beach situated upwind from the cement factory to compare with Ras Baridi to assess if heavy metal concentrations are associated with proximity to the factory and determine baseline levels for other rookeries on the Red Sea. In addition, more research should focus on assessing the heavy metal concentrations in the seagrass in these critical foraging habitats. A study on 16 female green turtles nesting at Ras Baridi revealed that the turtles migrate to multiple foraging areas in shallow coastal areas or in areas around offshore islands within the Red Sea basin (Al-Mansi et al., 2021). Further, as part of an ongoing study on post-nesting migrations patterns of green turtles tagged in Al Lith, one female was found to migrate to Ras Baridi (500 km distance) to forage (L Tanabe, 2022, unpublished data). Therefore, future work should also be conducted to assess the heavy metal concentration from the large seagrass meadows near Ras Baridi. Although information is lacking about sea turtles in this region, our study fills some of these knowledge gaps by providing concentrations of heavy metals from hatchlings at the largest green turtle rookery in the Red Sea.

## Conclusion

These data reported in this study present baseline levels of heavy metals (Cr, Mn, Fe, Co, Ni, Cu, Zn, As, Se, Cd, and Pb) from green turtle hatchlings’ liver, muscle, and residual yolk tissue at Ras Baridi, Saudi Arabia’s largest turtle rookery. To our knowledge, this is the first study on heavy metal concentrations in green sea turtles from the Red Sea region. This baseline data is critical for biomonitoring efforts in Saudi Arabia before major coastal development occurs. Over the next decade, under Vision 2030 (PIF, 2017), multiple giga-projects aim to accommodate more than two million people on the Red Sea coast in the next decade (PIF, 2017), increasing the potential of heavy metal input into the surrounding environments. Biomonitoring of sea turtles for heavy metal concentrations (and their nesting and feeding grounds) should be conducted as a long-term assessment of the impacts of these developments; the present study will allow for post-development comparison. Sea turtles are flagship species that have broad appeal and significance in ecotourism efforts, including in the nascent tourism industry in Saudi Arabia. Improved understanding of the potential heavy metal concentrations throughout the Saudi Arabian Red Sea can inform national turtle conservation efforts and identify areas requiring mitigation.

## Supplemental Information

10.7717/peerj.13928/supp-1 Supplemental Information 1 Supplementary methods and supplementary table.

10.7717/peerj.13928/supp-2 Supplemental Information 2 Raw data of heavy metal concentrations. Heavy metal concentration from hatchlings measured in the liver, muscle, and residual yolk of 22 hatchlings.

## Additional Information and Declarations

### Field Study Permissions

The following information was supplied relating to field study approvals (*i.e*., approving body and any reference numbers): Field experiments were approved by King Abdullah University of Science and Technology who obtained coast guard permits for the site.

### Data Availability

The following information was supplied regarding data availability: The raw measurements of heavy metals are available in the Supplemental Files.
