# Supplementary material for: An assessment of heavy metals in green sea turtle (Chelonia mydas) hatchlings from Saudi Arabia’s largest rookery, Ras Baridi
Source: PeerJ. 2022 Aug 23;10:e13928. doi: 10.7717/peerj.13928 (PMC9415434; doi:10.7717/peerj.13928)
Supplement: Supplemental Information 1 [file peerj-10-13928-s001.docx]

**An assessment of heavy metals in green sea turtle (*Chelonia mydas*) hatchlings from Saudi Arabia's largest rookery, Ras Baridi**

Lyndsey Kiana Tanabe^1^, Kirsty Scott^1^, Vijayalaxmi Dasari^1^, Michael Lee Berumen^1^

^1^Red Sea Research Center, Division of Biological and Environmental Science and Engineering, King Abdullah University of Science and Technology, Thuwal, 23955, Kingdom of Saudi Arabia

Corresponding Author:

Lyndsey Tanabe

Email address: [Lyndsey.Tanabe@kaust.edu.sa](mailto:Lyndsey.Tanabe@kaust.edu.sa)

Preparation of working solutions

1. Prepare 2% HNO3
   Sample dilution and carrier solution use 2 % HNO3. Add 2 mL concentrated HNO3

(≥ trace metal grade) into 98 mL of water to prepare 100 mL 2% HNO3.

1. Prepare 5% HNO3
   Instrument rinse uses 5% HNO3. To prepare 10 L of 5% HNO3, add 0.5 L nitric acid

(≥ trace metal grade) into 9.5 L DI water.

1. Prepare 1 mg/L internal standard

To prepare 20 mL internal standard, add 2 mL multi element standard solution 6020 ISS (4.1) into 18 mL 2% HNO3. The multi element standard solution 6020 ISS can be replaced by IV-ICPMS-71D standard solution (4.1).

1. Prepare calibration standards

Calibration standards containing the analytes of interest are prepared by diluting intermediate standard in 2% HNO3, with internal standard added at 40 μg/L.

Intermediate standard1 is 10 mg/L Inorganic Venture IV-ICPMS-71A multi element standard.

Intermediate standard 2 at 0.1 mg/L is prepared by dilution of 10 mg/L Inorganic Venture IV-ICPMS-71A multi element standard in 100 times with 2% HNO3.

Table S1. Calibration standards for analysis with Inductively Coupled Plasma Mass Spectrometer (ICP-MS).

| **Concentration**  **(μg/L)** | **Intermediate**  **Standard 1 (mL)** | **Intermediate**  **Standard 2 (mL)** | **10 mg/L Internal**  **Standard** | **2% HNO3 (mL)** |
| --- | --- | --- | --- | --- |
| 0 | 0 | 0 | 0.2 | 49.8 |
| 0.1 | 0 | 0.05 | 0.2 | 49.75 |
| 1 | 0 | 0.5 | 0.2 | 49.3 |
| 10 | 0.05 | 0 | 0.2 | 49.75 |
| 100 | 0.5 | 0 | 0.2 | 49.3 |
| 500 | 2.5 | 0 | 0.2 | 47.3 |

Table S2. Combined samples for analysis of each heavy metal (Cr, Mn, Fe, Co, Ni, Cu, Zn, As, Se, Cd, and Pb) from the yolk, muscle, and liver of 22 hatchlings. A minimum of 50 mg of dried tissue was needed for each analysis.

| **# hatchlings** | **Hatching ID(s)** | **Tissue** | **Beach** |
| --- | --- | --- | --- |
| 1 | DW1 | Yolk | Downwind |
| 1 | DW2 | Yolk | Downwind |
| 1 | DW3 | Yolk | Downwind |
| 1 | DW4 | Yolk | Downwind |
| 1 | DW5 | Yolk | Downwind |
| 1 | DW6 | Yolk | Downwind |
| 1 | DW7 | Yolk | Downwind |
| 1 | DW8 | Yolk | Downwind |
| 1 | DW9 | Yolk | Downwind |
| 1 | DW10 | Yolk | Downwind |
| 1 | DW11 | Yolk | Downwind |
| 1 | DW12 | Yolk | Downwind |
| 1 | DW13 | Yolk | Downwind |
| 1 | DW14 | Yolk | Downwind |
| 1 | DW15 | Yolk | Downwind |
| 1 | DW16 | Yolk | Downwind |
| 1 | F1 | Yolk | Factory |
| 1 | F2 | Yolk | Factory |
| 1 | F3 | Yolk | Factory |
| 1 | F4 | Yolk | Factory |
| 1 | F5 | Yolk | Factory |
| 1 | F6 | Yolk | Factory |
| 1 | DW1 | Muscle | Downwind |
| 2 | DW3, DW4 | Muscle | Downwind |
| 2 | DW5, DW6 | Muscle | Downwind |
| 2 | DW7, DW8 | Muscle | Downwind |
| 2 | DW9, DW10 | Muscle | Downwind |
| 2 | DW11, DW12 | Muscle | Downwind |
| 2 | DW13, DW14 | Muscle | Downwind |
| 2 | DW15, DW16 | Muscle | Downwind |
| 3 | F1, F2, F3 | Muscle | Factory |
| 3 | F4, F5, F6 | Muscle | Factory |
| 1 | DW1 | Liver | Downwind |
| 1 | DW3 | Liver | Downwind |
| 1 | DW4 | Liver | Downwind |
| 1 | DW5 | Liver | Downwind |
| 2 | DW6, DW7 | Liver | Downwind |
| 1 | DW8 | Liver | Downwind |
| 1 | DW9 | Liver | Downwind |
| 1 | DW10 | Liver | Downwind |
| 1 | DW11 | Liver | Downwind |
| 1 | DW12 | Liver | Downwind |
| 1 | DW13 | Liver | Downwind |
| 1 | DW14 | Liver | Downwind |
| 1 | DW15 | Liver | Downwind |
| 1 | DW16 | Liver | Downwind |
| 2 | F1, F2 | Liver | Factory |
| 2 | F3 | Liver | Factory |
| 1 | F4 | Liver | Factory |
| 1 | F5 | Liver | Factory |
| 1 | F6 | Liver | Factory |

**Table S3**. Method validation using a Certified Reference Material (ERM-CE278K). All the values are reported in mg/kg.

| **Element** | **Certified Value** | **Measured Value** | **Percentage of Recovery** |
| --- | --- | --- | --- |
| Cr | 0.73 ± 0.22 | 0.73 | 100.02 |
| Mn | 4.88 ± 0.24 | 4.24 | 91.32 |
| Fe | 161 ± 8 | 146.92 | 96.03 |
| Co | 0.21 | 0.19 | 90.48 |
| Ni | 0.69 ± 0.15 | 0.73 | 105.96 |
| Cu | 5.98 ± 0.27 | 5.50 | 96.26 |
| Zn | 71 ± 4 | 69.30 | 103.43 |
| As | 6.7 ± 0.4 | 5.91 | 93.86 |
| Se | 1.62 ± 0.12 | 1.57 | 96.63 |
| Cd | 0.336 ± 0.025 | 0.32 | 96.45 |
| Pb | 2.18 ± 0.18 | 1.92 | 96.02 |


Table S4. Summary of quality controls applied for metal analysis. All the values reported are in mg/kg.

| **Quality Control** | **Label** | **Cr** | **Mn** | **Fe** | **Co** | **Ni** | **Cu** | **Zn** | **As** | **Se** | **Cd** | **Pb** |
| --- | --- | --- | --- | --- | --- | --- | --- | --- | --- | --- | --- | --- |
| **Calibration blanks**  Prepared with the same solution that was used as a blank in the calibration of the equipment. Applied every 20 samples | call blank | 0.01 | 0.007 | 0 | 0.004 | 0.003 | 0 | 0.021 | 0.004 | 0.007 | 0.004 | 0.02 |
|  | call blank | 0.008 | 0.004 | 0 | 0.001 | 0 | 0 | 0 | 0.002 | 0.007 | 0.001 | 0.012 |
|  | call blank | 0 | 0.003 | 0 | 0.001 | 0 | 0 | 0.232 | 0 | 0.015 | 0 | 0.011 |
| **Matrix reaction blanks**  Prepared with the same digested solution of the samples and processed together with every batch of 20 samples | MRB1 | 0.064 | 0.013 | 0.464 | 0.014 | 0.085 | 0 | 1.494 | 0 | 0.015 | 0 | 0.009 |
|  | MRB2 | 0.166 | 0.01 | 0.12 | 0.001 | 0.451 | 0 | 1.015 | 0 | 0 | 0.002 | 0.003 |
|  | MRB3 | 0.029 | 0.007 | 0.224 | 0.002 | 0.105 | 0 | 1.067 | 0 | 0.022 | 0.001 | 0.003 |
|  | MRB4 | 0.045 | 0.055 | 0.078 | 0.001 | 0.107 | 0 | 0.39 | 0.001 | 0.011 | 0 | 0.001 |
| **Spiked blanks**  Blanks where spiked with 0.2 ml from 10 ppm STD(containing all the analytes) and diluted once every 20 samples | MFB1 | 36.453 | 38.038 | 36.954 | 37.135 | 36.978 | 36.949 | 37.482 | 36.275 | 36.895 | 38.034 | 38.096 |
|  | Recovery (%) | 91.02 | 94.958 | 92.19 | 92.835 | 92.178 | 92.373 | 92.73 | 90.685 | 92.209 | 95.085 | 95.2375 |
|  | MFB2 | 37.025 | 38.355 | 37.499 | 37.989 | 37.536 | 37.464 | 40.687 | 36.2335 | 36.177 | 37.478 | 40 |
|  | Recovery (%) | 92.45 | 95.75 | 93.553 | 94.97 | 93.573 | 93.66 | 100.743 | 90.5813 | 90.414 | 93.695 | 99.998 |
| **Duplicate samples**  Every 20 samples, one sample was randomly selected and analyzed by duplicate |  |  |  |  |  |  |  |  |  |  |  |  |
|  | RB-DW15 YOLK | 5.730 | 1.044 | 47.369 | 0.036 | 3.314 | 1.333 | 54.921 | 0.445 | 1.388 | 0.056 | 0.138 |
|  | RB-DW15YOLKDup | 5.745 | 1.040 | 47.065 | 0.041 | 3.289 | 1.281 | 54.623 | 0.444 | 1.473 | 0.066 | 0.134 |
|  | Difference (%) | 0.255 | 0.438 | 0.644 | 14.286 | 0.747 | 3.985 | 0.544 | 0.308 | 5.972 | 16.541 | 2.685 |
|  | RB-DW16MUSCLE | 52.050 | 3.483 | 377.847 | 0.491 | 31.829 | 3.921 | 82.226 | 0.481 | 1.024 | 0.006 | 0.147 |
|  | RB-DW16MUSCLEDup | 51.222 | 3.248 | 374.167 | 0.490 | 31.133 | 3.904 | 81.663 | 0.472 | 1.051 | 0.005 | 0.154 |
|  | Difference (%) | 1.603 | 6.995 | 0.979 | 0.203 | 2.211 | 0.433 | 0.688 | 1.883 | 2.596 | 18.182 | 4.651 |
|  | RBDW13LIVER | 0.721 | 3.169 | 472.901 | 0.029 | 0.620 | 3.221 | 39.745 | 0.372 | 2.066 | 0.006 | 0.044 |
|  | RBDW13LIVERDup | 0.691 | 3.311 | 455.243 | 0.026 | 0.564 | 3.169 | 39.041 | 0.379 | 2.110 | 0.004 | 0.041 |
|  | Difference (%) | 4.196 | 4.358 | 3.805 | 10.909 | 9.508 | 1.607 | 1.787 | 1.709 | 2.128 | 40.000 | 6.897 |
| **Spiked samples**  Every 20 samples, one sample was randomly selected and spiked with 0.2 ml from 10 ppm STD ( containing all the analytes) and diluted | RB F4 LIVER | 13.670 | 2.850 | 417.351 | 0.153 | 8.107 | 21.685 | 38.084 | 0.253 | 4.731 | 0.002 | 0.026 |
|  | RB F4 LIVER spike | 53.813 | 43.964 | 460.926 | 40.227 | 48.117 | 60.619 | 79.969 | 37.781 | 40.457 | 40.211 | 37.387 |
|  | Recovery (%) | 100.357 | 102.785 | 108.938 | 100.186 | 100.025 | 97.336 | 104.714 | 93.821 | 89.315 | 100.522 | 93.402 |
|  | RB-F6 YOLK | 6.868 | 1.076 | 52.102 | 0.177 | 4.034 | 1.893 | 51.311 | 0.132 | 1.592 | 0.049 | 0.096 |
|  | RB-F6 YOLK spike | 48.630 | 40.755 | 84.103 | 40.935 | 45.003 | 43.133 | 97.529 | 38.126 | 38.473 | 40.069 | 38.622 |
|  | Recovery (%) | 104.405 | 99.197 | 80.003 | 101.895 | 102.422 | 103.100 | 115.545 | 94.984 | 92.202 | 100.050 | 96.316 |
| **Continuous calibration verification** | CCV 1 | 51.468 | 49.021 | 49.839 | 49.333 | 49.431 | 49.587 | 50.721 | 47.7655 | 48.6725 | 50.477 | 48.458 |
| A solution of 50 μg/L was prepared containing all the analytes and measured every 20 samples in order to measure the validity of the calibration | Recovery (%) | 102.936 | 98.042 | 99.678 | 98.666 | 98.862 | 99.174 | 101.442 | 95.531 | 97.345 | 100.954 | 96.916 |
|  | CCV 2 | 51.415 | 48.149 | 50.288 | 50.388 | 51.167 | 50.681 | 50.727 | 47.6055 | 48.95 | 50.07 | 50.027 |
|  | Recovery (%) | 102.83 | 96.298 | 100.576 | 100.776 | 102.334 | 101.362 | 101.454 | 95.211 | 97.9 | 100.14 | 100.054 |
|  | CCV 3 | 52.421 | 49.115 | 49.281 | 50.595 | 51.354 | 51.204 | 51.272 | 48.0755 | 47.343 | 52.041 | 48.897 |
|  | Recovery (%) | 104.842 | 98.23 | 98.562 | 101.19 | 102.708 | 102.408 | 102.544 | 96.151 | 94.686 | 104.082 | 97.794 |
|  | CCV 4 | 53.214 | 51.375 | 49.872 | 50.869 | 50.905 | 51.307 | 51.381 | 48.012 | 47.287 | 51.847 | 50.117 |
|  | Recovery (%) | 106.428 | 102.75 | 99.744 | 101.738 | 101.81 | 102.614 | 102.762 | 96.024 | 94.574 | 103.694 | 100.234 |
|  | CCV 5 | 51.797 | 51.756 | 49.352 | 51.26 | 50.886 | 50.501 | 51.893 | 48.204 | 47.3095 | 51.242 | 48.485 |
|  | Recovery (%) | 103.594 | 103.512 | 98.704 | 102.52 | 101.772 | 101.002 | 103.786 | 96.408 | 94.619 | 102.484 | 96.97 |
|  | CCV 6 | 52.996 | 50.459 | 50.212 | 50.802 | 50.977 | 50.841 | 50.667 | 48.152 | 47.923 | 51.129 | 48.18 |
|  | Recovery (%) | 105.992 | 100.918 | 100.424 | 101.604 | 101.954 | 101.682 | 101.334 | 96.304 | 95.846 | 102.258 | 96.36 |
|  | CCV 7 | 53.101 | 51.729 | 49.438 | 50.383 | 50.324 | 50.411 | 51.079 | 47.2395 | 46.6145 | 50.67 | 48.052 |
|  | Recovery (%) | 106.202 | 103.458 | 98.876 | 100.766 | 100.648 | 100.822 | 102.158 | 94.479 | 93.229 | 101.34 | 96.104 |
|  | CCV 8 | 51.585 | 50.843 | 49.76 | 50.468 | 50.758 | 50.797 | 50.747 | 47.29 | 46.428 | 51.09 | 49.279 |
|  | Recovery (%) | 103.17 | 101.686 | 99.52 | 100.936 | 101.516 | 101.594 | 101.494 | 94.58 | 92.856 | 102.18 | 98.558 |
|  | CCV 9 | 51.582 | 54.103 | 48.731 | 50.063 | 50.472 | 50.097 | 50.727 | 46.928 | 46.217 | 51.322 | 48.416 |
|  | Recovery (%) | 103.164 | 108.206 | 97.462 | 100.126 | 100.944 | 100.194 | 101.454 | 93.856 | 92.434 | 102.644 | 96.832 |
|  | CCV 10 | 51.209 | 50.227 | 49.88 | 50.955 | 51.141 | 50.938 | 51.485 | 47.508 | 46.307 | 50.689 | 49.892 |
|  | Recovery (%) | 102.418 | 100.454 | 99.76 | 101.91 | 102.282 | 101.876 | 102.97 | 95.016 | 92.614 | 101.378 | 99.784 |
| **Second vendor verification** | QCS1 | 107.788 | 98.203 | 105.341 | 106.97 | 105.909 | 110.502 | 107.69 | 98.444 | 96.309 | 106.998 | 101.917 |
| A solution of 100 μg/L containing all the analytes was prepared from a second vendor and measured one every 20 samples. | Recovery (%) | 107.788 | 98.203 | 105.341 | 106.97 | 105.909 | 110.502 | 107.69 | 98.444 | 96.309 | 106.998 | 101.917 |
|  | QCS2 | 106.773 | 99.941 | 104.034 | 109.074 | 104.417 | 106.483 | 105.082 | 100.73 | 97.285 | 104.923 | 105.732 |
|  | Recovery (%) | 106.773 | 99.941 | 104.034 | 109.074 | 104.417 | 106.483 | 105.082 | 100.73 | 97.285 | 104.923 | 105.732 |

Table S5. Results from the Mann-Whitney U-tests computed for each heavy metal comparing concentrations between the Factory and Downwind beaches. Cd could not be computed with ties.

|  | W | *p* |
| --- | --- | --- |
| Cr | 220 | 0.57 |
| Mn | 246 | 0.99 |
| Fe | 267 | 0.68 |
| Co | 175 | 0.12 |
| Ni | 225 | 0.65 |
| Cu | 167 | 0.09 |
| Zn | 292 | 0.34 |
| As | 472 | < 0.01 |
| Se | 217 | 0.53 |
| Cd | - | - |
| Pb | 322 | 0.11 |

Table S6. Results from the Kruskal-Wallis tests computed for each heavy metal comparing concentrations between tissues sampled (liver, muscle, and yolk).

|  | X^2^ | *p* |
| --- | --- | --- |
| Cr | 18.59 | < 0.01 |
| Mn | 36.85 | < 0.01 |
| Fe | 39.65 | < 0.01 |
| Co | 13.41 | < 0.01 |
| Ni | 17.73 | < 0.01 |
| Cu | 35.43 | < 0.01 |
| Zn | 26.22 | < 0.01 |
| As | 8.32 | 0.02 |
| Se | 33.60 | < 0.01 |
| Cd | 27.45 | < 0.01 |
| Pb | 11.90 | < 0.01 |
